# Amylin receptor agonism enhances the effects of liraglutide in protecting against the acute metabolic side effects of olanzapine

**DOI:** 10.1016/j.isci.2023.108628

**Published:** 2023-12-04

**Authors:** Kyle D. Medak, Stewart Jeromson, Annalaura Bellucci, Meagan Arbeau, David C. Wright

**Affiliations:** 1 Department of Human Health and Nutritional Sciences, University of Guelph, Guelph, ON N1G 2W1, Canada; 2 School of Kinesiology, University of British Columbia, Vancouver, BC V6T 1Z1, Canada; 3 British Columbia Children’s Hospital Research Institute, Vancouver, BC V5Z 4H4, Canada; 4 Faculty of Land and Food Systems, University of British Columbia, Vancouver, BC V6T 1Z4, Canada

**Keywords:** Drugs, Pharmacology, Physiology, Molecular biology

## Abstract

Olanzapine is a second-generation antipsychotic (AP) used in the management of schizophrenia. Although effective at reducing psychoses, APs cause rapid hyperglycemia, insulin resistance, and dyslipidemia, an effect mediated in part by glucagon. We tested if amylin, a hormone that reduces glucagon, or the amylin receptor agonist pramlintide would protect against acute olanzapine-induced impairments in glucose and lipid homeostasis alone or in combination with other glucose-lowering agents such as liraglutide. We demonstrated that pramlintide lowered olanzapine-induced increases in glucagon:insulin ratio with a trend to protect against excursions in blood glucose. There was an additive effect of pramlintide and liraglutide in protecting against olanzapine-induced hyperglycemia, which was mirrored by reductions in glucagon and attenuated markers of dyslipidemia. Our findings provide evidence that pramlintide, although moderately protective against some aspects of olanzapine-induced metabolic dysfunction, can be used to enhance the protective effects of other interventions against acute olanzapine-induced metabolic dysfunction.

## Highlights

• Olanzapine reduces circulating amylin concentrations
• Pramlintide and liraglutide additively reduce olanzapine-induced hyperglycemia
• This “cocktail” also improves olanzapine-induced dyslipidemia
• A “cocktail” approach should be considered to reduce side effects of AP drugs

## Graphical abstract

**Figure:**
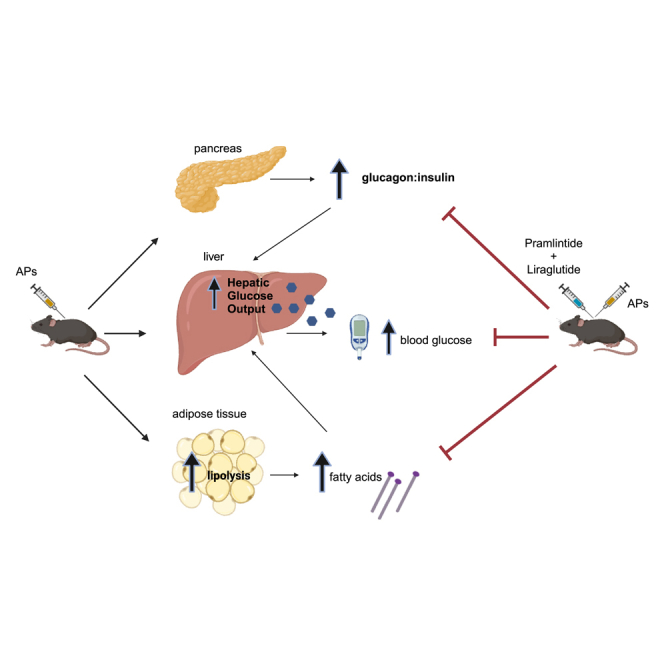

## Introduction

Schizophrenia is a debilitating mental illness affecting ∼1% of the population.^1^ Antipsychotic drugs (APs) such as olanzapine are commonly used in the treatment of schizophrenia and a wide list of on- and off-label disorders such as anxiety, sleep disorders, and attention deficit disorder.^2^ This treatment approach is successful in reducing symptoms of psychosis through inhibition of the dopamine (D2), serotonin (5-HT2A), and muscarinic (M3) receptors, among others, but has severe effects on metabolic health, which are likely impacted by both central and peripheral mechanisms, including hyperglycemia, dysregulated lipid metabolism, weight gain, development of type 2 diabetes, and increased risk of mortality.^3^^,^^4^^,^^5^^,^^6^^,^^7^^,^^8^^,^^9^

Metabolic impairments caused by APs had initially been attributed to weight gain; however, there is now a growing appreciation of weight-gain-independent effects on glucose and lipid homeostasis. For instance, a single dose of AP is sufficient to cause impairments in carbohydrate and fat metabolism in rodents,^10^^,^^11^^,^^12^^,^^13^^,^^14^^,^^15^^,^^16^ findings that have been recapitulated in humans in whom weight-gain-independent effects of APs have been noted.^17^^,^^18^ Our group has provided strong evidence that acute olanzapine-induced hyperglycemia is mediated by increases in glucagon. In support of this, we have shown that acute olanzapine treatment increases circulating glucagon,^10^^,^^11^^,^^13^ whereas protection against olanzapine-induced hyperglycemia is often paralleled by reductions in glucagon and/or the glucagon:insulin ratio.^10^^,^^12^ Perhaps most convincingly, the hyperglycemic effects of olanzapine are absent in glucagon receptor knockout mice, despite the development of profound insulin resistance.^19^ These acute excursions in blood glucose, which are observed with each treatment with olanzapine, can be harmful as they increase the risk for the development of oxidative stress, inflammation, and cardiometabolic disease.^20^^,^^21^ It is important to point out that acute AP-induced impairments to glucose homeostasis do not weaken over time and are still potent after repeated treatments in rodents.^16^^,^^22^

Amylin (also known as islet amyloid polypeptide [IAPP]) is a peptide hormone that is co-secreted with insulin from the pancreatic beta cell in response to nutrient stimuli.^23^^,^^24^ Treatment with amylin analogs has been employed to exert key functions on glycemic control in patients with diabetes, including inhibition of glucagon secretion.^25^ Conversely, lack of amylin signaling increased glucagon secretion^26^^,^^27^ and led to a greater glycemic response following a glucose challenge.^26^ It is unclear if olanzapine exerts acute effects on amylin secretion though olanzapine treatment inhibits secretion of its partner hormone, insulin.^28^ In this investigation, we aimed to determine if (1) olanzapine impacts circulating amylin, (2) exogenous amylin analogs would be protective against olanzapine-induced impairments in glucose and lipid metabolism, and (3) there are synergistic effects of combining amylin analogs and other glucose-lowering drugs such as the GLP1 receptor agonist liraglutide. We hypothesized that amylin and the amylin receptor agonist pramlintide would confer protection against olanzapine-induced perturbations in glucose homeostasis and that these effects would be additive with liraglutide.

## Results

### Serum amylin is lower in olanzapine-treated animals and negatively correlated with blood glucose AUC

**Figure 1:**
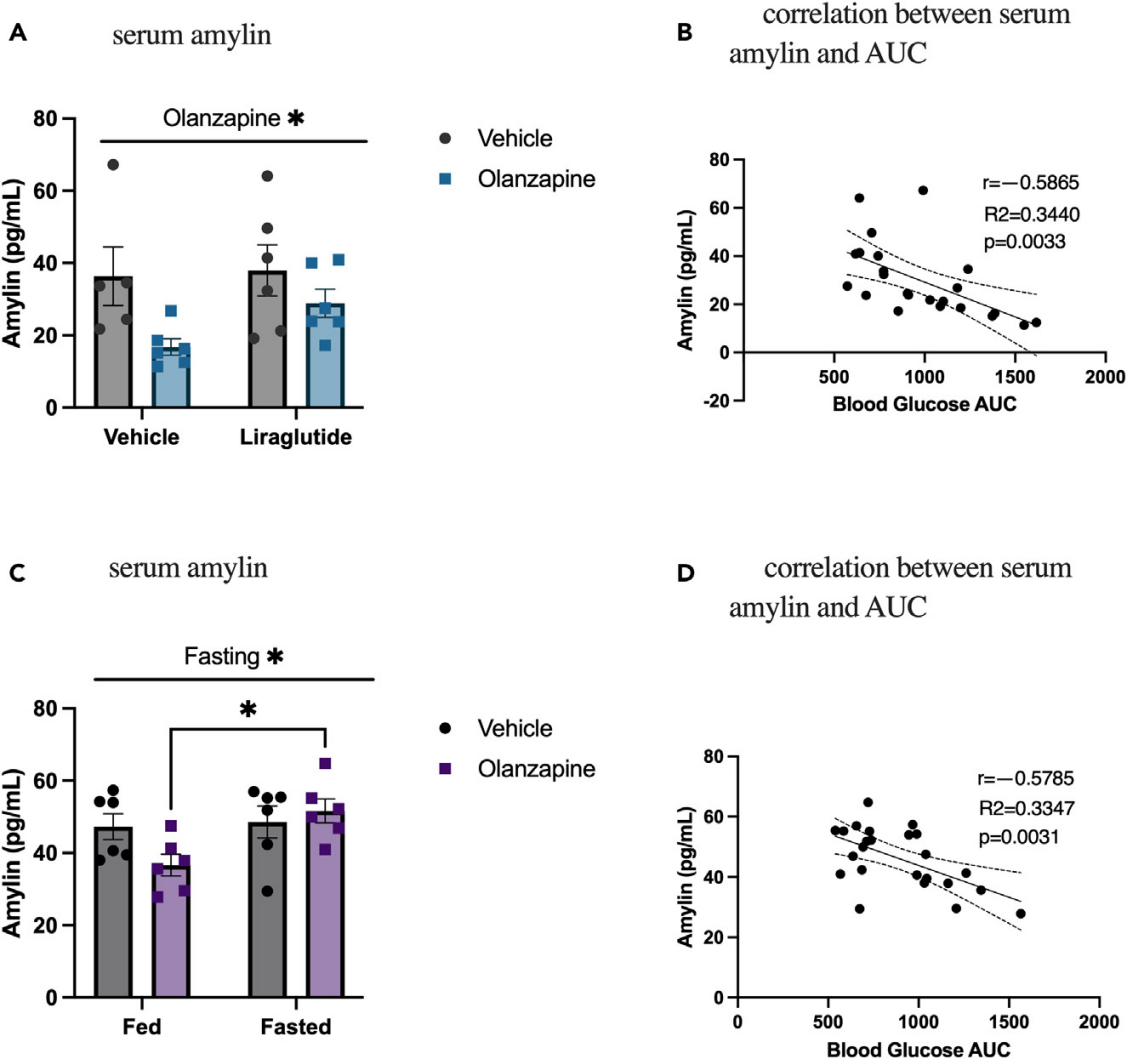
Serum amylin is lower in olanzapine-treated animals and negatively correlated with blood glucose AUC (A) Male mice were co-treated with IP injections of olanzapine (5 mg/kg) and liraglutide (400 μg/kg) for 120 min, and amylin was measured from serum collected via cardiac puncture (n = 5–6 mice/group). (B) Blood glucose area under the curve (AUC) over 120-min treatment was calculated (n = 23) and correlated to serum amylin after 120 min with displayed linear regression line and 95% confidence bands. (C) Male mice were fasted for 16 h then treated with an IP injection of olanzapine (5 mg/kg) for 120 min, and amylin was measured from serum collected via cardiac puncture (n = 6 mice/group). (D) Blood glucose area under the curve (AUC) over 120-min treatment was calculated (n = 24) and correlated to serum amylin after 120 min with displayed linear regression line and 95% confidence bands. Serum amylin was analyzed by two-way ANOVA. Line of best fit was determined by simple linear regression. A line above the graph indicates a significant main effect; ∗p < 0.05 between indicated groups. Data are presented as mean ± SEM. Picogram (pg); milliliter (mL).

To determine if olanzapine affects circulating amylin, we measured serum amylin 2 h following treatment with olanzapine and/or a high dose of liraglutide (400 μg/kg IP). Liraglutide treatment in these same animals completely protected against olanzapine-induced hyperglycemia^12^ and allowed us to examine serum amylin across a range of glucose responses to olanzapine. Olanzapine-treated animals had lower serum amylin (Figure 1A), and this effect was not impacted by liraglutide treatment. We then correlated serum amylin and the blood glucose AUC (Figure 1B) (AUC data published in^12^) and found that there was a significant negative correlation between blood glucose AUC and serum amylin.

To further explore the relationship between amylin- and olanzapine-induced hyperglycemia, we measured serum amylin concentrations in mice that had *ad libitum* access to standard rodent chow or had been fasted for 16 h prior to the olanzapine challenge. In these same mice, we have previously reported that fasting protected against olanzapine-induced increases in blood glucose.^13^ As shown in Figure 1C, serum amylin concentrations were increased by fasting, and when analyzed for multiple comparisons by Tukey’s post-hoc analysis, the olanzapine-induced reduction in amylin is prevented by fasting. Moreover, in these fasted mice, there was a significant negative correlation between the glucose AUC (data published in^13^) and serum amylin (Figure 1D). These findings suggest that treatment with amylin or amylin analogs might confer protection against olanzapine-induced hyperglycemia.

### Amylin treatment does not protect against olanzapine-induced hyperglycemia or lipidemia

**Figure 2:**
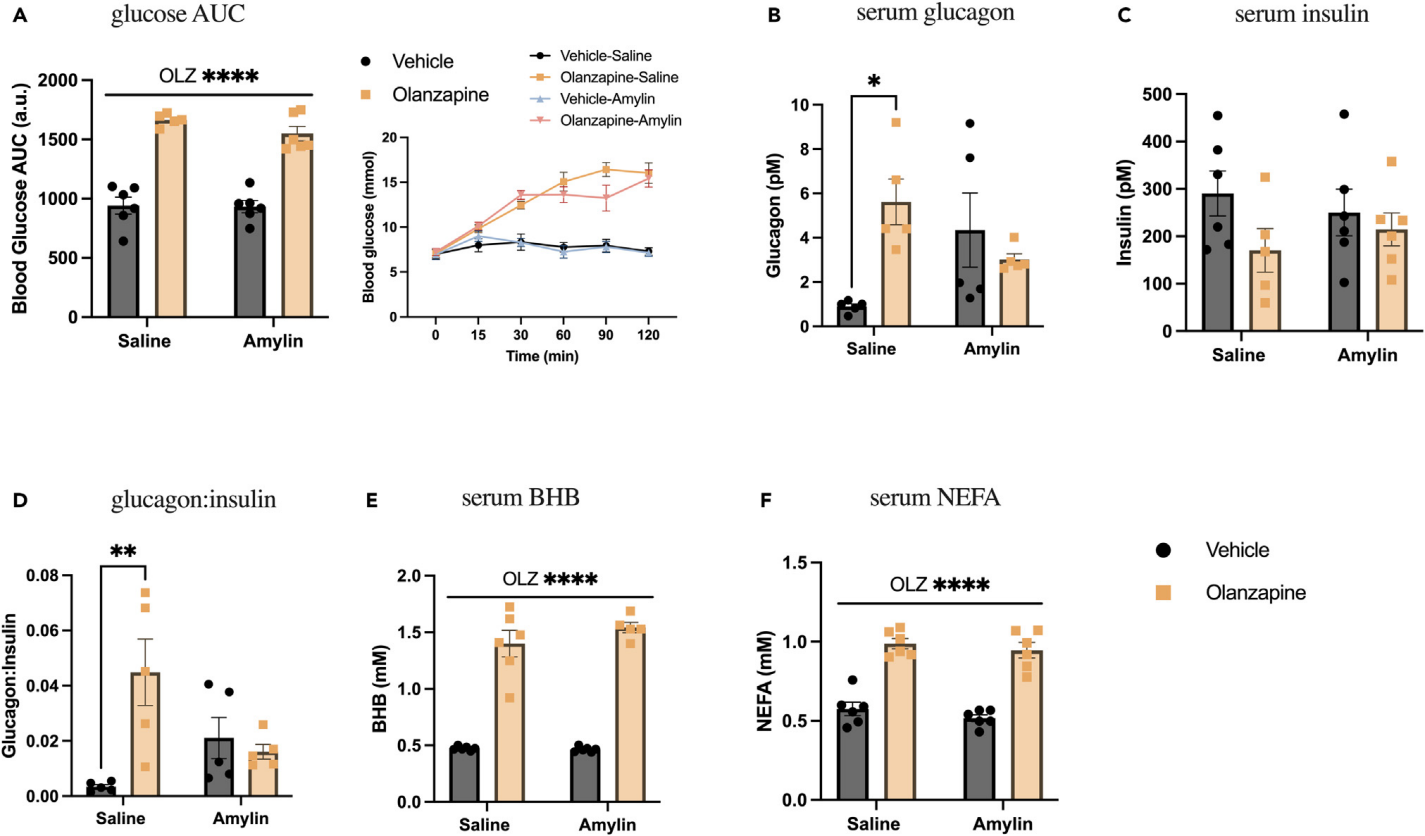
Amylin treatment does not protect against olanzapine-induced hyperglycemia or lipidemia Male mice were cotreated with an IP injection of olanzapine (5 mg/kg) and subcutaneous amylin (2 μg/mouse) for 120 min. (A) Blood glucose was measured from the distal tail blood and area under the curve (AUC) calculated (n = 5–6 mice/group). Following 120 min of olanzapine and amylin cotreatment, glucagon (B), insulin (C), the ratio of glucagon:insulin (D), beta-hydroxybutyrate (E), and non-esterified fatty acids (F) were measured in serum. Blood glucose AUC and serum hormones/metabolites were analyzed by two-way ANOVA. Lines over graphs indicate a main effect of the described parameter, whereas bars connected by lines indicate a significant difference between indicated groups. ∗p < 0.05, ∗∗p < 0.01, ∗∗∗∗p < 0.0001. All data are presented as mean ± SEM. Picomolar (pM); millimolar (mM).

As circulating amylin levels were negatively correlated with olanzapine-induced hyperglycemia, we tested if amylin cotreatment would offset the metabolic side effects of olanzapine. To accomplish this, we treated mice with olanzapine (5 mg/kg IP) and amylin (2 μg/mouse SQ) at the same time. Blood glucose AUC was increased by olanzapine but unchanged by amylin treatment (Figure 2A). The olanzapine-induced increase in glucagon was not present in amylin-treated animals (Figure 2B); however, amylin alone appeared to increase glucagon, and there was no change in insulin (Figure 2C), resulting in a glucagon:insulin ratio that was increased by olanzapine in vehicle-treated animals and unchanged in amylin-treated animals (Figure 2D). Consistent with our group’s previous work,^10^^,^^11^^,^^12^^,^^13^^,^^16^ there was an effect of olanzapine to increase serum beta-hydroxybutyrate (BHB) (Figure 2E), a marker of whole-body fat oxidation,^29^ and non-esterified fatty acids (NEFA) (Figure 2F), and these endpoints were not impacted by cotreatment with amylin. These data demonstrate that treating with amylin is not sufficient to protect against olanzapine-induced metabolic disturbances and that pharmacological manipulation of amylin signaling may be required to blunt the negative metabolic side effects of acute olanzapine treatment.

### Pramlintide treatment protects against olanzapine-induced glucose dysregulation but not lipidemia

**Figure 3:**
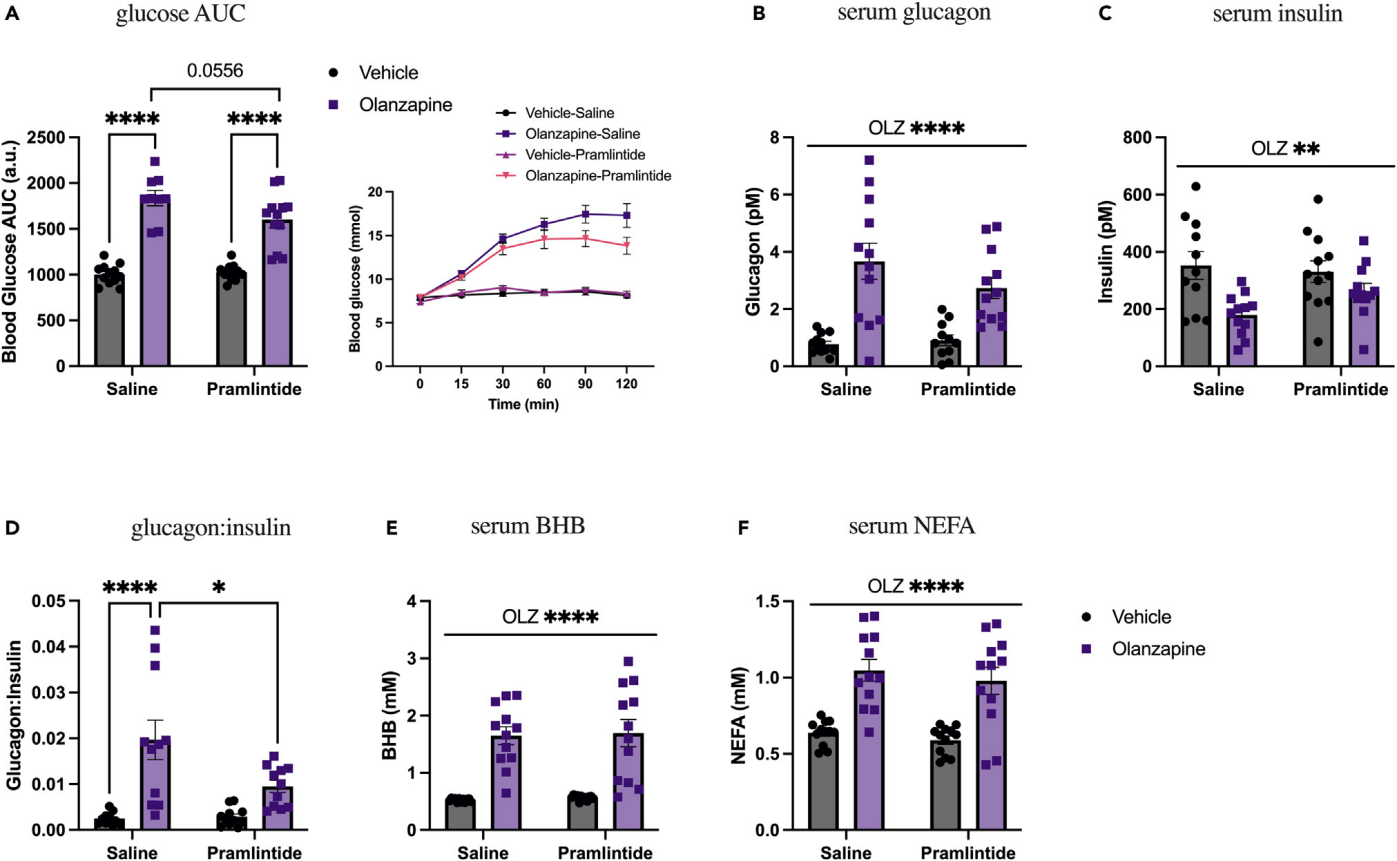
Pramlintide treatment trends toward protection against olanzapine-induced glucose dysregulation but not lipidemia Male mice were co-treated with an IP injection of olanzapine (5 mg/kg) and subcutaneous pramlintide (1 μg/mouse) for 120 min. (A) Blood glucose was measured from the distal tail blood and area under the curve (AUC) calculated (n = 9–12 mice/group). Following 120 min of olanzapine and pramlintide cotreatment, glucagon (B), insulin (C), the ratio of glucagon:insulin (D), beta-hydroxybutyrate (E), and non-esterified fatty acids (F) were measured in serum. Blood glucose AUC and serum hormones/metabolites were analyzed by two-way ANOVA. Lines over graphs indicate a main effect of the described parameter, whereas bars connected by lines indicate a significant difference between indicated groups. ∗p < 0.05, ∗∗p < 0.01, ∗∗∗∗p < 0.0001. All data are presented as mean ± SEM. Picomolar (pM); millimolar (mM).

We next aimed to determine if the pharmacological agonist of the amylin receptor, pramlintide, was able to protect against metabolic side effects of olanzapine. We cotreated mice at the same time with olanzapine (5 mg/kg) and pramlintide (1 μg/mouse), as this dose of pramlintide has previously been shown to reduce blood glucose excursions in rodents in response to a bolus of glucose.^30^ The higher blood glucose AUC in olanzapine-treated animals strongly trended to be lower with pramlintide cotreatment (p = 0.0556; Figure 3A), i.e., the olanzapine-induced rise in blood glucose (45.4%) was ∼25% less when cotreated with pramlintide (35.9%) at the same time. There was a main effect of olanzapine to increase glucagon (Figure 3B) and reduce insulin (Figure 3C), and the olanzapine-induced increase in the glucagon:insulin ratio was blunted when pramlintide was co-treated with olanzapine (Figure 3D). The serum markers of lipid oxidation, BHB (Figure 3E), lipidemia, and NEFA (Figure 3F) were elevated in olanzapine-treated animals and unaffected by pramlintide. Here, we find that pramlintide can be moderately protective against some aspects of olanzapine-induced metabolic dysfunction.

To explore if we can gain an enhanced benefit from amylin signaling by allowing pramlintide to exert its effects prior to olanzapine treatment (i.e., lactate peak is approximately 30 min postinjection^30^^,^^31^), we also tested an alternative timing of treatment. We pretreated with pramlintide 30 min prior to olanzapine and then measured the blood glucose response. Pramlintide pretreatment had no effect on any blood or serum measurements including blood glucose AUC, serum insulin, glucagon, or the ratio of glucagon:insulin (Figure S1), suggesting that adjusting treatment timing does not provide further benefit to the acute effects of olanzapine. Furthermore, it is unknown if olanzapine modifies the effective dose needed to protect against excursions in blood glucose. We tested a much higher dose of amylin (5 μg/mouse) or pramlintide (5 μg/mouse) at the same time as olanzapine (5 mg/kg) to determine if even higher pharmacological doses would be effective in protecting against olanzapine-induced hyperglycemia. We observed no difference in blood glucose AUC between mice treated with olanzapine or cotreated with olanzapine and amylin (5 μg/mouse) or pramlintide (5 μg/mouse) when analyzed by an ordinary one-way ANOVA (Figure S2).

### Pramlintide enhances the effects of low-dose liraglutide on olanzapine-induced hyperglycemia and lipidemia

**Figure 4:**
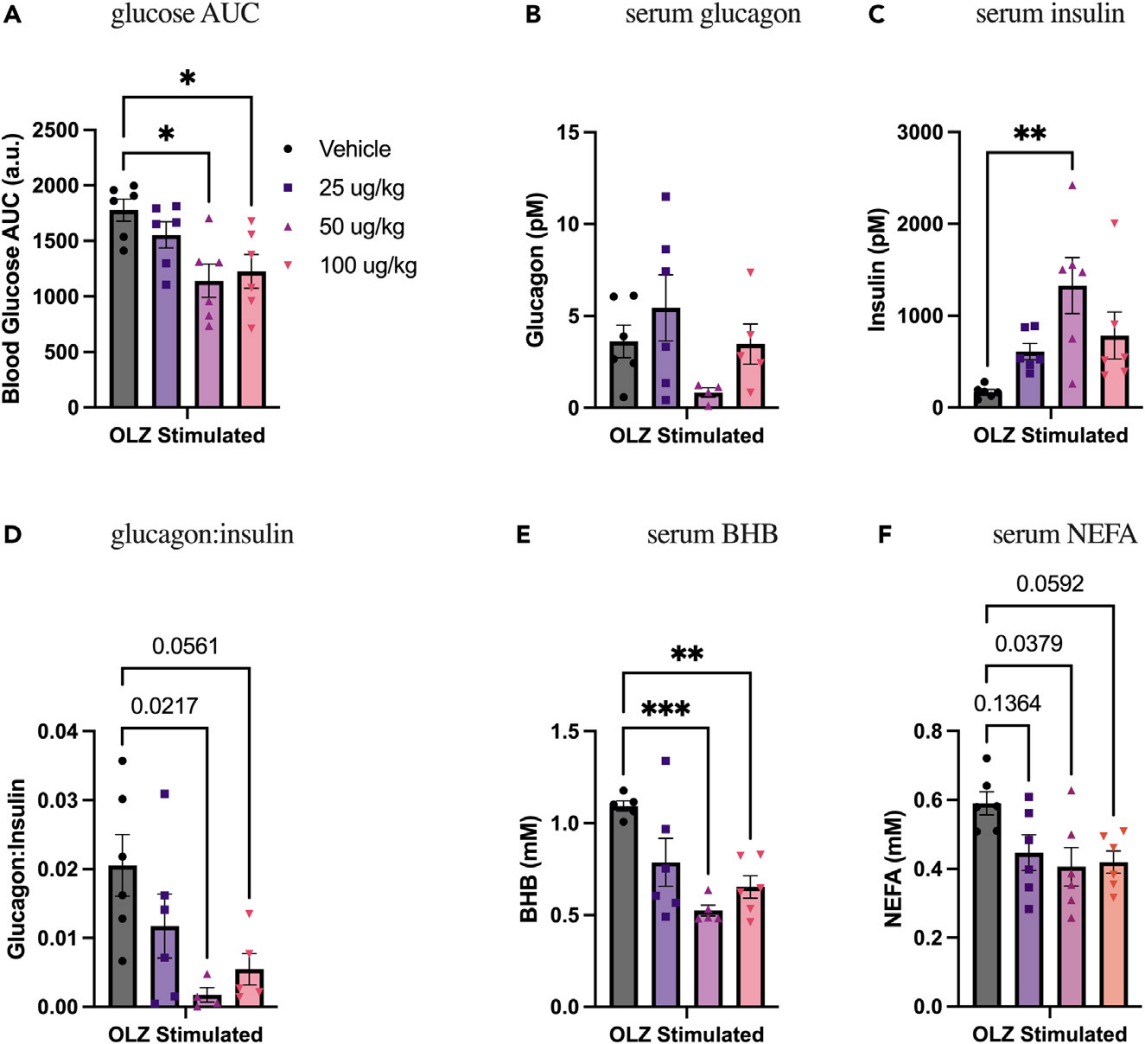
Dose response of liraglutide under olanzapine-stimulated conditions Male mice were cotreated with IP injections of olanzapine (5 mg/kg) and liraglutide for 120 min. (A) Blood glucose area under the curve (AUC) over 120-min treatment was calculated (n = 6). Following 120 min of olanzapine and liraglutide, glucagon (B), insulin (C), the ratio of glucagon:insulin (D), beta-hydroxybutyrate (E), and non-esterified fatty acids (F) were measured in serum. Blood glucose AUC and serum hormones/metabolites were analyzed by ordinary one-way ANOVA with multiple comparisons, and bars connected by lines indicate a significant difference between indicated groups. ∗p < 0.05, ∗∗p < 0.01, ∗∗∗p < 0.001, and p values are represented in areas of interest. All data are presented as mean ± SEM. Picomolar (pM); millimolar (mM).

Given that there can be issues with tolerability and dose escalation with the high dose of liraglutide used in our previous work (400 μg/kg; Figure 1),^32^ we performed a dose response of liraglutide under olanzapine-stimulated conditions (Figure 4). We did this to determine if a lower dose of liraglutide, which did not protect against metabolic side effects of olanzapine, would have synergistic/additive effects when combined with pramlintide. We found that 25 μg/kg liraglutide, treated at the same time as olanzapine, did not protect against olanzapine-induced hyperglycemia (Figure 4A), changes in glucoregulatory hormones (Figures 4B–4D), BHB (Figure 4E), or NEFA (Figure 4F), whereas higher doses did. Based on these results this low-dose liraglutide was used in subsequent pramlintide-liraglutide cotreatment experiments.

**Figure 5:**
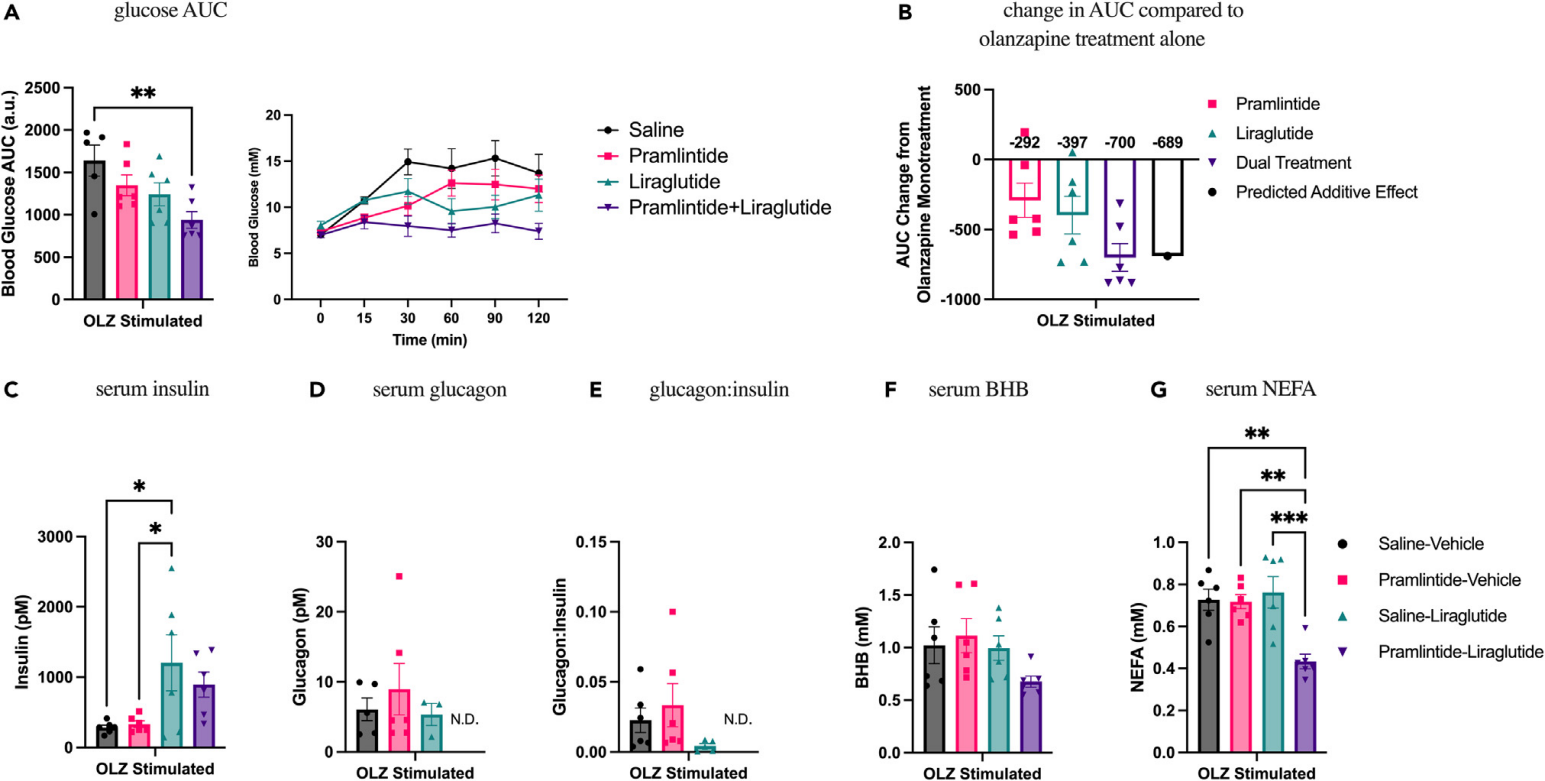
Pramlintide can enhance the effects of low-dose liraglutide on olanzapine-induced hyperglycemia and lipidemia Male mice were treated with IP injections of olanzapine (5 mg/kg) and/or liraglutide (25 μg/kg) and/or an SQ injection of pramlintide (1 μg/mouse) for 120 min. (A) Blood glucose was measured from the distal tail blood and area under the curve (AUC) calculated (n = 4–6 mice/group). (B) The change in AUC from olanzapine monotreatment was calculated for all co-treatment groups. Following 120 min of olanzapine, pramlintide, and/or liraglutide co-treatment, glucagon (C), insulin (D), the ratio of glucagon:insulin (E), beta-hydroxybutyrate (F), and non-esterified fatty acids (G) were measured in serum. Blood glucose AUC and serum hormones/metabolites were analyzed by ordinary one-way ANOVA with multiple comparisons, and bars connected by lines indicate a significant difference between indicated groups. ∗p < 0.05, ∗∗p < 0.01, ∗∗∗p < 0.001. All data are presented as mean ± SEM. Group values that were below detection are labeled as not detected (N.D.). Picomolar (pM); millimolar (mM).

Mice were treated with olanzapine (5 mg/kg) alone or in combination with pramlintide (1 μg/kg) and/or liraglutide (25 μg/kg). We found that under olanzapine-stimulated conditions, animals that received dual liraglutide and pramlintide treatment had significantly reduced blood glucose AUC compared with untreated mice (Figure 5A) and that when the predicted difference compared with olanzapine monotreatment is calculated, the dual treatment had an equivalent effect to the combined effects of each treatment alone (Figure 5B). Serum insulin was increased in animals that had been treated with liraglutide (Figure 5C), whereas glucagon (Figure 5D) and glucagon:insulin ratio (Figure 5E) were below detection in animals that received dual treatment. Serum BHB was unchanged between all olanzapine-stimulated cotreatment groups (Figure 5F); however, we found a synergistic effect of pramlintide and liraglutide to reduce NEFA, whereas there was no effect of each treatment alone (Figure 5G). Overall, we show that pramlintide and liraglutide have an additive effect to blunt olanzapine-induced increases in blood glucose AUC and a synergistic effect to reduce lipidemia.

## Discussion

Antipsychotic drugs can result in rapid perturbations in glucose and lipid metabolism that occur independent of changes in body weight and adiposity.^11^^,^^14^^,^^15^^,^^16^^,^^33^^,^^34^ In the current investigation, we provide evidence that combination treatment with low-dose liraglutide and pramlintide protects against olanzapine-induced hyperglycemia.^10^

The acute effects of APs on blood glucose are likely mediated by increases in glucagon. In support of this, we have found that olanzapine-induced hyperglycemia is absent in glucagon-receptor-deficient mice, whereas interventions that dampen acute olanzapine-induced hyperglycemia such as wheel running, fasting, and ketogenic diets are mirrored by a blunted increase in glucagon following olanzapine treatment.^10^^,^^11^^,^^12^^,^^13^^,^^19^^,^^35^ Consistent with this, we were unable to detect serum glucagon following olanzapine treatment in mice that had received a “cocktail” of liraglutide and pramlintide.

Amylin analogs have been employed as effective pharmacological tools to improve glycemic control in diabetic patients, in part, by inhibiting glucagon secretion.^25^ In the current investigation, we show a significant negative correlation between amylin and blood glucose AUC during olanzapine treatment and an attenuation in the glucose and glucagon response to olanzapine in mice co-treated with pramlintide. Pramlintide and amylin have been described to have similar potency in lowering postprandial glucose and glucagon^36^ but we do not observe this relationship. The differing effects between these molecules when cotreated with olanzapine, at least at the time point in which metabolites are analyzed, could perhaps arise due to differences in clearance. Rodent amylin has a reported half-life of approximately 13 min,^37^ whereas pramlintide has a plasma half-life of approximately 50 min^38^ when administered by SQ injection. This may also explain why treatment at the same time as olanzapine is more effective than pretreatment with pramlintide 30 min prior to olanzapine (Figure S1), as the effects of pramlintide are likely lost before the peak of olanzapine-induced hyperglycemia.

GLP1 exhibits similar effects as amylin to regulate hyperglycemia, in part, through blunting of inappropriate glucagon secretion, for example post-prandially in individuals with diabetes^24^ or as shown in the present study with olanzapine-induced hyperglycemia. GLP1, unlike amylin, also has effects on insulin secretion^39^ and can completely protect against olanzapine-induced hyperglycemia.^12^ In our recent paper using GLP1 analogs with olanzapine, we have shown that liraglutide treatment (400 μg/kg) negates olanzapine-induced increases in glucose, glucagon, and glucagon:insulin ratio, whereas a GLP1 receptor antagonist potentiated olanzapine-induced increases in glucose and glucagon.^12^ In clinical practice, the effect of GLP1 receptor agonists is attenuated over time, particularly for long-acting agents, commonly leading to dose escalation and intensifying adverse side effects.^32^ We reasoned that the effect of pramlintide cotreatment to lower serum glucagon:insulin ratio could be used alongside lower doses of liraglutide to enhance protection from olanzapine-induced hyperglycemia and enable lower doses of drugs to be used.

There is support for targeting the combination of amylin and GLP1 signaling pathways, as this intervention has been shown to benefit glucose regulation, satiety, and body weight while improving treatment tolerability.^40^^,^^41^^,^^42^ Although both GLP1 and amylin are released in response to a meal, and exert regulatory effects on blood glucose and satiety, they do so through different mechanisms and sites of action, possibly allowing for even greater effects by combining treatment with these hormones. While the GLP1 receptor is expressed in the gut, the brain, and the exocrine pancreas,^43^ the expression of the amylin receptor on the other hand is largely confined to the central nervous system^44^ and exerts effects on peripheral glucoregulatory control via descending vagal pathways.^45^ In support of this, there are synergistic effects of GLP1 receptor agonists and amylin receptor agonists to reduce food intake in non-human primates.^46^ Furthermore, the development of peptide hybrids producing signaling action on both GLP1 and amylin receptors produce greater glucose control and weight loss than each agonist alone.^47^ In line with these data, in the current investigation we show that the use of amylin receptor and GLP1 receptor agonists at the same time has an additive effect on suppressing olanzapine-induced hyperglycemia. We believe that this is likely due to distinct actions of these drugs on peripheral and central mechanisms regulating glucagon secretion.

A commonly overlooked consequence of acute AP treatment is the impact on lipid homeostasis. We, and others, have shown that acute olanzapine treatment results in robust increases in serum fatty acids, markers of adipose tissue lipolysis, fat oxidation, and accumulation of triglyceride in the liver within 2 h.^10^^,^^11^^,^^13^^,^^48^^,^^49^ It had been initially hypothesized that this increase in fat delivery and oxidation could be a mechanism through which APs induce insulin resistance, but this has been refuted. In a study designed by Klingerman and colleagues,^49^ mice were cotreated with olanzapine and an inhibitor of fatty acid oxidation, etomoxir.^50^ After this cotreatment, mice quickly required euthanasia and did not survive the intervention, providing evidence that olanzapine-induced increases in fat oxidation are likely not the cause of insulin resistance but rather a consequence of it, as the rapid switch to fat oxidation is required for sufficient provision of energy substrates in the absence of glucose oxidation.^49^ Within this context, our findings might suggest that the reduction in serum fatty acids with dual liraglutide and pramlintide treatment might be secondary to changes in glucose homeostasis.

Findings from the current study show that the amylin receptor agonist pramlintide protects against olanzapine-induced glucose dysregulation but not lipidemia. We build upon previous work demonstrating that high-dose liraglutide fully protects against olanzapine-induced metabolic dysfunction^12^ and use pramlintide to enhance lower dose liraglutide to protect against metabolic side effects of olanzapine. Our findings suggest that a “cocktail” approach should be considered when developing treatment strategies with AP drugs and that future translational work could benefit from the combination of amylin and GLP1 receptor agonists as an adjunct treatment to lessen the acute metabolic consequences of APs.

### Limitations of study

We have previously shown that female mice are protected against acute AP-induced hyperglycemia.^10^ Given this, only male mice were examined in the current investigation and thus this somewhat limits the translational relevance of the current findings. Furthermore, it should be addressed that our data do not show a reduction in amylin following a 16-h fast (Figure 1C), though fasting is known to decrease insulin and, at the same time, amylin.^51^ This amylin measurement occurred following a 2-h acute olanzapine experiment during which the stress associated with acute injections of either olanzapine or vehicle could have resulted in increases in blood glucose and commensurate increases in amylin. Also, during this time, even control mice would not be eating their food, and it is possible that this could have reduced the difference in amylin between groups.

It should be noted that in addition to acute metabolic side effects, APs such as olanzapine also cause marked weight gain when given chronically.^52^^,^^53^ Future studies are required to determine if there are beneficial effects of combined pramlintide and liraglutide therapy in mitigating the long-term metabolic complications of APs.

## STAR★Methods

### Key resources table

**Table:** 

REAGENT or RESOURCE	SOURCE	IDENTIFIER
**Chemicals, peptides, and recombinant proteins**		

Olanzapine	Cayman Chemicals	Cat# 11937-25
Dimethyl sulfoxide (DMSO)	Sigma-Aldrich	Cat# 34869
Kolliphor EL	Sigma-Aldrich	Cat# 5135
Liraglutide	Cayman Chemicals	Cat# 24727
Amylin (rat, mouse)	Cayman Chemicals	Cat# 24275-500
Pramlintide (acetate hydrate)	Cayman Chemicals	Cat# 24093-5

**Critical commercial assays**		

Mouse IAPP/Amylin (Competitive EIA) ELISA Kit	Cayman Chemicals	Cat# LS-F9888-1
Glucagon ELISA	Mercodia	Cat# 10-1281-01
Mouse Insulin ELISA	Mercodia	Cat# 10-1247
β-Hydroxybutyrate (Ketone Body) Colorimetric Assay Kit	Cayman Chemicals	Cat# 700190
Non-Esterified Fatty acid (NEFA) – HR Series NEFA (Color Reagent A and B, Solvent A and B)	FugiFilm Wako Diagnostics	Cat# 999–34691; 991–34891; 995–34791; 993-35191

**Experimental models: Organisms/strains**		

Mouse C57BL/6J	Jackson Laboratories	Cat# 000664; RRID:IMSR_JAX:000664
Software and algorithms		
GraphPad Prism 9	GraphPad Software	https://www.graphpad.com/

**Other**		

S-2335 Mouse Breeder Sterilizable Diet	Teklad Diets Harlan Laboratories	Cat# 7004

### Resource availability

#### Lead contact

Further information and requests for resources and reagents should be directed to and will be fulfilled by the lead contact, David C. Wright (david.wright@ubc.ca).

#### Materials availability

This study did not generate new unique reagents. Blood glucose test strips and a Freestyle Lite handheld glucometer were acquired from Abbott Diabetes Care Inc. (Alameda, CA, USA). Injections were carried out using 25-gauge needles purchased from ThermoFisher Scientific (Mississauga, ON, CAN). ELISAs obtained from Mercodia Inc. (Winston-Salem, NC 27103, USA) were used to measure serum glucagon and insulin. Kits used to measure Amylin/IAPP and Beta-Hydroxybutyrate were obtained from Cayman Chemicals (Ann Arbor, MI, USA). Serum non-esterified fatty acid (NEFA) (Wako Bioproducts, Richmond, VA, USA) was measured on 96-well plates as per the manufacturer’s instructions. ELISAs and assays were performed in duplicate with a standard curve and samples were loaded in single when serum was limited.

#### Data and code availability

(1) All data reported in this paper will be shared by the lead contact upon request.
(2) This paper does not report original code.
(3) Any additional information required to reanalyze the data reported in this paper is available from the lead contact upon request.

### Experimental model and study participant details

#### Animals

All experimental procedures were approved by the University of Guelph and University of British Columbia Animal Care Committees and followed Canadian Council on Animal Care guidelines. Approximately 10-week-old male C57BL/6J mice were purchased from Jackson Laboratories (Bar Harbor, ME) and group housed in clear polycarbonate shoebox-style cages (dimensions: 7 1⁄2″ x 11 1⁄2″ x 5″) with wire lids. Rooms were kept at an ambient temperature of 22°C with 45% humidity and a 12:12 h light dark cycle. Animals were given free access to water and standard rodent chow (7004-Teklad S-2335 Mouse Breeder Sterilizable Diet; Teklad Diets Harlan Laboratories, Madison WI). Mice were given ∼2-3 weeks to acclimate to the facilities before experimentation. All olanzapine experiments occurred at the beginning of the animals’ light cycle which coincides with the clinical recommendation for drug administration prior to bedtime.^54^

### Method details

#### Terminal drug co-treatment tolerance tests

Olanzapine was dissolved in DMSO (1 mg/100 μL) to create a stock solution. Kolliphor EL solution and saline (500 μL/900 mL) were used to dilute 500 μL of the stock olanzapine solution and mice were injected intraperitoneally (IP) with olanzapine (5 mg/kg) or vehicle (DMSO, Kolliphor EL, saline) at the beginning of the light cycle (∼0900). Drug and vehicle were prepared from powdered drug and stored stock solutions (DMSO, Kolliphor EL, and saline) each experimental day. We^19^^,^^35^ and others^55^ have previously used this dose as it mimics human dosing requirements based on dopamine-binding occupancy in rats given olanzapine by subcutaneous injections.^56^ For fasting experiments, animals were moved to a new cage, with or without food, for 16 h then treated with olanzapine (5 mg/kg) for a 2-h period at the beginning of the light cycle (∼0900). For high dose liraglutide co-treatment experiments, animals were treated with olanzapine (5 mg/kg) and/or liraglutide (400 μg/kg) at the same time and blood glucose was tracked for a 2-h period. In amylin (2ug/mouse) and pramlintide (1ug/mouse) experiments, these powdered stocks were dissolved in saline and injected subcutaneously (SQ).^30^^,^^57^ DMSO was used to dilute liraglutide (1 mg/mL) to create a stock solution then diluted in saline and injected IP at 400, 100, 50, or 25 μg/kg. Combinations of these drugs were administered alongside olanzapine (5 mg/kg) or, in some cases, 30 min prior to olanzapine. Blood glucose was measured in mice prior to, 15-, 30-, 60-, 90-, and 120-min post-drug administration using a handheld glucometer sampled from a drop of blood taken from the tail vein using a distal tail snip. At 120 min post olanzapine treatment mice were anesthetized with sodium pentobarbital (5 mg/100 g body weight) then cardiac blood was collected with 25-gauge needles, allowed to clot for ∼20 min at room temperature, and centrifuged at 5000g for 10 min at 4°C. Serum was stored at −80°C until further analysis.

### Quantification and statistical analysis

Statistical tests were completed using GraphPad Prism v.9.0 (GraphPad Software, La Jolla, CA, USA). The effects of drug co-treatment on glucose AUC and serum measures were analyzed by two-way ANOVA, followed by Tukey’s post-hoc analysis if there was a significant interaction between drug treatments in which case the p *value* of the discussed group comparison is represented (∗p < 0.05, ∗∗p < 0.01, ∗∗∗p < 0.001, ∗∗∗∗p < 0.0001 between indicated groups). If we failed to find a significant interaction between groups, main effects were displayed above graphs. Experiments in which ‘dual treatment’ with pramlintide and/or liraglutide were given and all animals were treated with olanzapine were analyzed by ordinary one-way ANOVA followed by Tukey’s post-hoc analysis for multiple comparisons, in which pairwise comparisons are represented on the graph (∗p < 0.05, ∗∗p < 0.01, ∗∗∗p < 0.001, ∗∗∗∗p < 0.0001 between indicated groups). Correlational data was analyzed by 2-tailed Pearson correlation with 95% confidence interval and displayed line of best fit from simple linear regression. Datasets were analyzed for outliers with Grubbs’ test using Graphpad Outlier Calculator and values were excluded if identified as outliers. Glucose curves were displayed but not statistically analyzed as this information was represented in AUC values. Normality was assessed using the Shapiro-Wilk test unless a sample size was large enough to use the D’Agostino & Pearson test, as per the recommendation of Graphpad Statistics Guide. A relationship was considered significant when p < 0.05.
