# Supplementary material for: Amylin receptor agonism enhances the effects of liraglutide in protecting against the acute metabolic side effects of olanzapine
Source: iScience. 2023 Dec 4;27(1):108628. doi: 10.1016/j.isci.2023.108628 (PMC10767228; doi:10.1016/j.isci.2023.108628)
Supplement: Document S1. Figures S1 and S2 [file mmc1.pdf]

iScience, Volume *27*

Supplemental information

Amylin receptor agonism enhances the effects of liraglutide in protecting against the acute metabolic side effects of olanzapine

Kyle D. Medak, Stewart Jeromson, Annalaura Bellucci, Meagan Arbeau, and David C. Wright

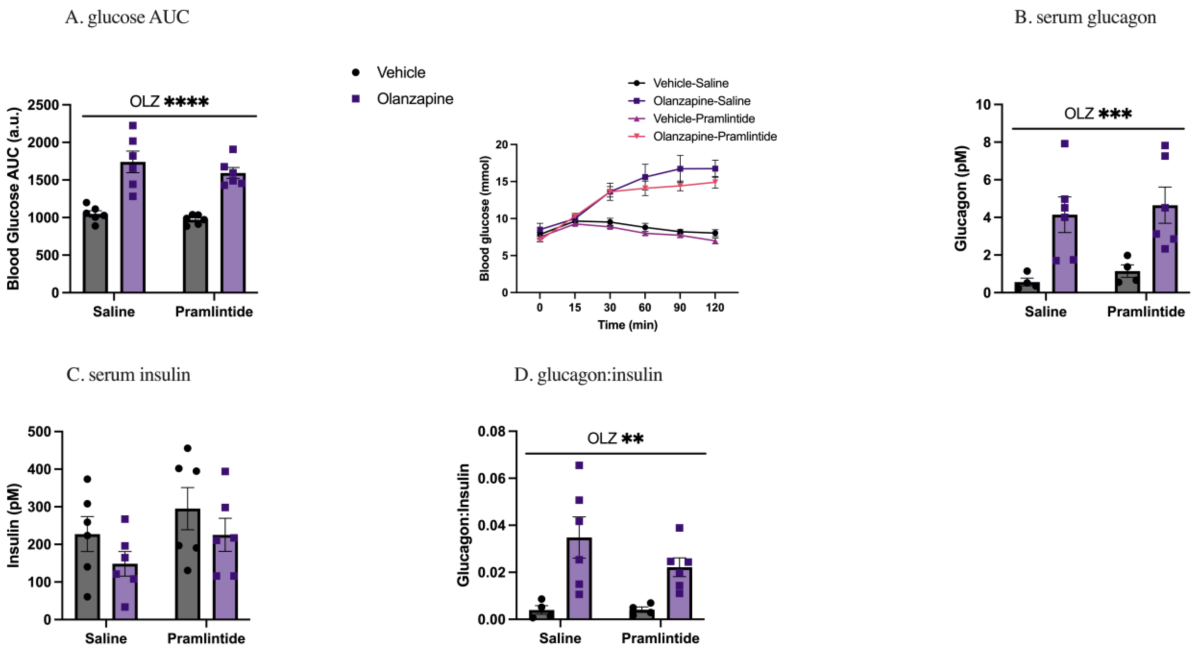

Supplemental Figure 1: Pramlintide pre-treatment does not protect against olanzapine-induced hyperglycemia, related to Figure 3. Male mice were co-treated with an IP injection of olanzapine (5 mg/kg) for 120 minutes and/or SQ injection of pramlintide (1 ug/mouse) 30 minutes prior to olanzapine. (**A**) Blood glucose was measured from the distal tail blood and area under the curve (AUC) calculated (n=6 mice/group). Following olanzapine and pramlintide treatment, glucagon (**B**), insulin (**C**), and the ratio of glucagon:insulin (**D**). Blood glucose AUC and serum hormones/metabolites were analyzed by two-way ANOVA. Lines over graphs indicate a main effect of the described parameter. **P < 0.01, ***P < 0.001, ****p<0.0001. All data are presented as mean ± SEM.

A. glucose AUC

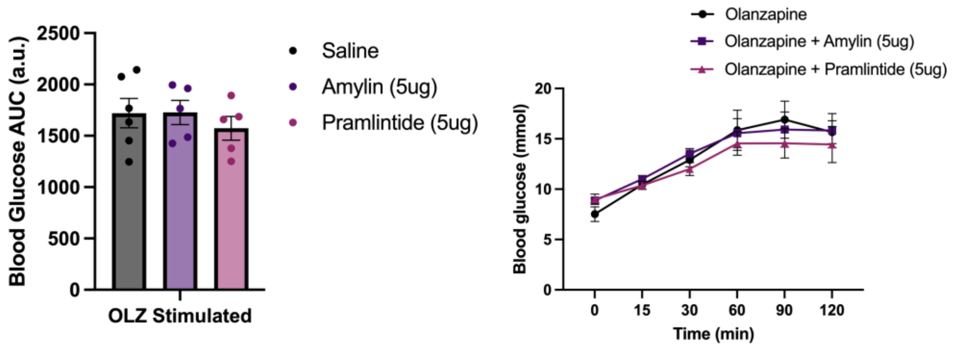

Supplemental Figure 2: Higher dose amylin or pramlintide does not protect against olanzapine-induced hyperglycemia, related to Figures 2 and 3. Male mice were co-treated with an IP injection of olanzapine (5 mg/kg) for 120 minutes and SQ injection of amylin (5 ug/mouse) or pramlintide (5 ug/mouse). (**A**) Blood glucose was measured from the distal tail blood and area under the curve (AUC) calculated (n=5-6 mice/group). Blood glucose AUC was analyzed by ordinary one-way ANOVA with multiple comparisons and bars connected by lines would indicate a significant difference between indicated groups. All data are presented as mean ± SEM.
